# Trial of Acute Femoral Fracture Fixation (TrAFFix): study protocol for a randomised controlled feasibility trial

**DOI:** 10.1186/s13063-017-2265-0

**Published:** 2017-11-14

**Authors:** Xavier L. Griffin, Matthew L. Costa, Juul Achten, Melina Dritsaki, Janis Baird, Nicholas Parsons

**Affiliations:** 1 OxfordTrauma, NDORMS, Kadoorie Centre, University of Oxford, Oxford, OX3 9DU UK; 2 NDORMS, University of Oxford, Windmill Road, Oxford, OX3 7LD UK; 3 MRC Lifecourse Epidemiology Unit, Southampton General Hospital, University of Southampton, Southampton, SO16 6YD UK; 4 Statistics and Epidemiology Unit, Warwick Medical School, University of Warwick, Coventry, CV4 7AL UK

**Keywords:** Femur, Randomised controlled trial, Fracture fixation

## Abstract

**Background:**

Distal femoral fractures are a source of considerable morbidity and best treatment is currently uncertain. The Trial of Acute Femoral Fracture Fixation (TrAFFix) is a randomised, parallel-group feasibility study designed to inform the design of a later, definitive clinical trial comparing intramedullary nails and locking plates for the treatment of distal femoral fractures.

**Methods/design:**

Patients aged 50 years and older with a femoral fracture within the distal two Müller squares are potentially eligible for inclusion. Participants are randomly allocated to receive fixation with either an intramedullary nail or a distal locking plate. Measurements (EuroQol 5 Dimensions, Dementia Quality of Life, Disability Rating Index) are collected at baseline, 6 weeks and 4 months. The recruitment rate will be assessed across seven participating centres over a total of 52 centre-months which is expected after 10 months of recruitment. Objectives are – feasibility phase, to assess recruitment rate and completion rate of the primary outcome measure; process evaluation, to assess the generalisability and likely success of a future trial; definitive trial, quantify and draw inferences on observed differences in health-related quality of life at 4 months between the study intervention groups (nail versus plate). A favourable opinion was granted by the Wales Research Ethics Committee (16/WA/0225), study-wide NHS approval was given by the Health Research Authority (IRAS 206745), and participating NHS trusts provided local approvals. This study was funded by the National Institute for Health Research Health Technology Assessment (HTA 15/59/22).

**Discussion:**

This is the protocol for a feasibility study conducted prior to any future definitive trial. The estimates of participant recruitment rate and proportion of data completion will be coupled with outputs from the process evaluation to make a final decision regarding feasibility

**Trial registrations:**

The study is registered with the National Institute for Health Research Portfolio (CPMS ID: 32536) and the ISRCTN registry (ISRCTN92089567) on 26 May 2016.

## Background

Fractures of the distal femur are increasingly common injuries. They account for 5% of all fractures of the femur with an estimated incidence of 10 per 100,000 [1]. The optimal management of these fractures remains controversial. There is a bimodal distribution of the incidence of these fractures with age [2]. The majority, approximately 85%, are fragility fractures sustained by older-aged patients after a fall from a standing height, the remainder are typically sustained by multiply injured patients after massive trauma [2].

There has been very little research exploring treatment options for distal femoral fractures in this population. A recent Cochrane review [3] found few trials in this area, most of which compared outdated implants, such as non-anatomic, non-locking plating systems or earlier-generation nails. Furthermore, important limitations in the methodology of each of the trials were identified leading to substantial risks of bias. It was suggested that in order to optimise patient functional recovery following this debilitating injury ‘a well-designed, adequately powered, randomised controlled trial comparing modern treatments is required’ [4].

It is current practice to manage displaced fractures with operative fixation if the patient is medically fit enough to undergo surgery [5, 6]. Surgery reduces the substantial complications associated with non-operative treatment, such as prolonged immobilisation and bed rest, as well as the problems of non-union and mal-union [7].

Since the operative treatment of these fractures was popularised, there has been a wide variety of implants employed to achieve fixation. The two interventions most commonly used in UK practice are intramedullary fixation with a locked, retrograde nail (nail), and extramedullary fixation with an anatomical, angular stable plate (locking plate) [5]. Nails offer twin theoretical advantages; the mechanical impact of a long, intramedullary device that is close to the axis of the femur [8] and the biological advantages of minimum disruption of the fracture site and stimulation of blood supply through reaming [9]. However, nails provide only limited options for distal locking screws, as all screws must pass through the centre of the nail, so the stability of the bone-implant construct may be sub-optimal. Locking-plate fixation has been facilitated by recent advances in implant technology that allow the screws to be screwed into the bone as well as the plate itself (‘locked’). This produces a ‘fixed-angle’ bone-plate construct. These plates were designed specifically for use in osteoporotic bone, and have been shown to exhibit excellent biomechanical properties [10]. However, they are more expensive than nails and require larger surgical wounds to apply.

There are few clinical data available to guide clinicians [4], and it is clear that there is no current consensus concerning the best management of these injuries [5]. We performed a multicentre retrospective study to review the current management of distal femoral fractures at four UK major trauma centres [5]. We found that only two devices are now used for fixation – retrograde nails and locking plates. This retrospective study illustrates the patient demographics, the variability in treatment of these fractures in the UK and the considerable morbidity associated with the injury.

Some studies suggest that there may be an important difference in outcomes following the choice of surgical management of these patients. The mean benefit of a nail over locking plates may be as great as 0.12 in EQ-5D-derived utility scores (*p* = 0.019) [11]. The minimum clinically important difference for EQ-5D is estimated to be 0.08 [12]. Similar effect sizes are demonstrated in other measures of function and quality-of-life, such as the Glasgow Outcome Scale (extended) and the 12-item Short Form Health Survey (SF-12) [11, 13, 14]. These findings have also been reported by other groups. A small pilot study in the US comparing these technologies found some evidence of a similar benefit in quality of life in favour of nails (mean difference EQ-5D 0.1, *p* = 0.07) [15].

We propose to conduct a feasibility study for a later, definitive randomised controlled trial comparing functional outcome after treatment with modern intramedullary nails or anatomical locking plates for fractures of the distal femur.

### Objectives

The objectives of the TrAFFix study are to:

1. Assess the feasibility of a future definitive trial
2. Perform a process evaluation to understand the generalisability and likely success of a future trial

## Methods/design

### Study design summary

This study is a multicentre, two-arm, parallel-group, individual-randomised controlled feasibility study comparing outcomes following locking-plate fixation with intramedullary nail fixation in the treatment of patients with fractures of the distal femur.

A process evaluation will be performed in parallel with the feasibility study. This evaluation will use a mixed-methodology approach. This will include qualitative interviews with staff and participants as well as a quantitative assessment of the characteristics of the sample, the fidelity of the interventions and the acceptability of the follow-up schedule.

This protocol is prepared in accordance with Standard Protocol Items: Recommendations for Interventional Trials (SPIRIT) guidance whose Figure and Checklist can be found in Table 1 and the appended file, respectively.

**Table 1:** Schedule of enrolment, interventions and assessments

	Study period			
	Enrolment	Allocation	Post allocation	
Time point	0	0	6 weeks	4 months
Enrolment				
Eligibility:				
Fracture classification [31]	X			
Cognitive impairment	X	X		
Age (≥50 years)	X			
Informed consent/agreement	X			
Intervention				
Intervention A		X		
Intervention B		X		
Additional fixation		X		
Anaesthesia		X		
Grade of surgeon		X		
Prescribed medications		X		
Rehab assessment		X		
Assessments				
Baseline characteristics:				
Contact details				
Date of birth		X		
Sex		X		
Current medications		X		
Comorbidities		X		
Current/previous occupation		X		
Educational attainment		X		
Grip strength		X	X	X
Self-efficacy report		X		
Rockwood Frailty Scale score		X		
MOS social support		X		
Government benefits		X		
Residential status		X		
Discharge destination		X		
Mobility		X		
Outcomes				
EQ-5D-5 L		Pre + post injury	X	X
DEMQoL^a^		Pre + post injury	X	X
DRI^b^		Pre + post injury	X	X
Radiographs		X	X	
Complications		X	X	X
Health economics				X
Qualitative interviews^c^		X	X	X

^a^Patients with chronic cognitive impairment, ^b^Patients without chronic cognitive impairment, ^c^Selection of patients and staff

*DEMQoL* Dementia Quality of Life, *DRI* Dementia Rating Index, *EQ-5D-5 L* EuroQol 5 Dimensions (5 L) Score *MOS* Medical Outcomes Survey

### Settings

Participants were recruited from six NHS hospitals in England. These sites represented a diverse group of hospitals ranging from tertiary-referral-level-1 trauma centres to non-specialist district hospitals.

### Eligibility

#### Feasibility study

All adult patients presenting at the trial centres with an isolated, acute, fracture of the distal femur are vpotentially eligible to take part in the trial. Fragility fractures will be defined as those sustained by adults aged 50 years and over [16]. Patients were initially eligible for this study if they:

- Are aged 50 years or older as a surrogate for bone density and, therefore, fragility fracture
- Have a fracture of the femur involving the distal two ‘Müller’ squares [17]
- Would, in the opinion of the attending surgeon, benefit from internal fixation of the fracture

During recruitment, after review of the screening data from the trial centres, it became apparent that substantial numbers of patients were being excluded due to the age cut-off. The Trial Steering Committee recommended that the 50 years of age boundary be removed to test the feasibility of recruiting a broader population.

Patients will be excluded from this study if they have:

- A loose knee or hip arthroplasty requiring revision
- Pre-existing femoral deformity
- An arthroplasty that precludes nail fixation

Patients with chronic cognitive impairment or acute delirium, any comorbidities, and open and closed distal femoral fractures will be included. For patients with bilateral fractures of the femur, only one fracture will be included in the study.

### Process evaluation

All patients who are eligible for inclusion in the feasibility study and their personal consultees, as well as all staff members involved in the research and intervention delivery, will be eligible to be approached about participating in qualitative interviews as part of the process evaluation.

### Consent

The nature of these injuries means that the great majority of patients will be operated on immediately or on the next available trauma operating list, depending on access to an appropriate operating theatre. Some patients may be unconscious, all will be distracted by the injury to their lower limb and its subsequent treatment and all will have had large doses of opiates for pain relief, potentially affecting their ability to process information. Similarly, patients’ next-of-kin, carers and friends are often anxious at this time and may have difficulty in weighing the large amounts of information. In this emergency situation the focus is on obtaining consent for surgery (where possible) and informing the patient and any next-of-kin about immediate clinical care.

The consent procedure for this trial will reflect that of the surgery, with the clinical team assessing capacity before taking consent for the surgical procedure and this capacity assessment then being used to decide on the proper approach to consenting to the research. Conducting research in this ‘emergency setting’ is regulated by the *Mental Capacity Act 2005* (MCA). We propose to act in accordance with section 32, subsection 9b of the MCA following a process approved by the relevant research ethics committee; where the clinical team advise that prospective patient consent is appropriate, this will be sought by the research team. If the clinical team advise that prospective patient consent is not appropriate, the research team will approach an appropriate consultee.

Where a personal consultee is available, they will be provided with the study information and their written agreement recorded where given. Where a personal consultee is not available then an appropriate nominated consultee will be identified to advise the research team. The nominated consultee will be asked to agree for the patient to be randomised; this agreement will be prospectively recorded during the electronic randomisation process.

For those patients who did not prospectively consent, or who had a nominated consultee give prospective agreement, the research associate will provide the patients with all of the study information at the first appropriate time when the patient has regained capacity. For those patients with capacity who did not consent prior to surgery and still lack capacity after their surgery, a personal consultee will be contacted to advise the research team about the patient’s continued participation in the study. Written consent or agreement for continued participation in the study will be recorded.

As part of the initial consent process patients and their personal consultees will be asked whether they may be approached about participating in interviews with regards their views on participating in this trial. A researcher from the TrAFFix team will identify which patients will be approached for interview, from those who agree to be approached. Participants who agree to be approached for interview will have a separate informed consent discussion for the interviews with a member of the research team either in person, by phone, or by post and where given their written or oral consent will be recorded. Conversations between researchers and patients or personal consultees may be audio-recorded as part of the process evaluation. This may include the initial discussion about TrAFFix before informed consent for the main study has been give. Where audio-recording may be acceptable and appropriate, local research staff will explain the rationale for the process evaluation and recording of the conversation and ask patients whether the conversation can be audio-recorded. Where patients agree, their verbal consent will be recorded at the start of the recording.

Responsibility for recording and dating both oral and written informed consent or agreement will be with the investigator, or persons delegated by the investigator, who conducted the informed consent discussion. If a participant withdraws from the study, data collected up until the point of withdrawal will be included in any subsequent analyses.

### Randomisation and blinding

Patients will be enrolled and randomised using a secure online system provided by the Oxford Clinical Trials Research Unit. Randomisation will be on a 1:1 basis, stratified by participating hospital and by whether, in the opinion of the clinical care team, the patient has chronic cognitive impairment. Participants will be randomly allocated to fixation using either intramedullary nailing or locking-plate fixation.

As the surgical scars are clearly visible the patients cannot be formally blinded to their treatment, but will only be informed of their allocated treatment if requested at the end of the trial. The treating surgeon will not be blind to the treatment allocation, but will take no part in the post-operative assessment of outcomes.

### Treatment pathway

#### Standardised treatment pathway

Participants will usually be assessed in the emergency department. Diagnosis of a fracture of the distal femur will be confirmed from plain radiographs of the femur. Where there is doubt over the radiological pattern of the fracture; for example, whether it extends into the knee or not, participants will be reviewed by the on-call orthopaedic surgeon and where clinically indicated a computed tomography (CT) may be performed – this constitutes standard of care practice.

All participants will undergo the following investigations as a minimum: electrocardiogram, full blood count, group and save, coagulation screen, urea, creatinine and electrolytes. Routine thromboprophylaxis will be started in all participants not already receiving anticoagulant therapy. Pharmaceutical and mechanical prophylaxis measures will be used in accordance with current practice agreed at each centre. A regional or general anaesthesia technique will be used and routine analgesia provided according to local practice.

All participants will receive perioperative prophylactic antibiotics in accordance with current practice agreed at each centre. Appropriate preparation, positioning and fracture reduction will be left to the discretion of the operating surgeon, as per their normal clinical practice.

#### Treatment options

##### Intramedullary nailing

Fixation of the fracture will be achieved with a proximally and distally locked nail that spans the entire diaphysis of the femur. All nails will be introduced retrograde through the knee joint. In this pragmatic trial, the details of surgical incision and approach, fracture reduction and supplementary fixation with wires or screws will be at the surgeon’s discretion as per their normal clinical practice.

##### Locking-plate fixation

Fixation of the fracture will be achieved with an anatomical, distal-femoral locking-plate and screws. Locking plates will be defined as those in which at least one fixed-angle locking screw is placed distal to the fracture. The operating surgeon will determine the length, number and type of additional screws. Additional fixation with lag screws and cerclage wires will be at the surgeon’s discretion. In this pragmatic trial, the details of surgical incision and approach, fracture reduction, number and type of other screws and supplementary fixation with wires or screws will be at the surgeon’s discretion as per their normal clinical practice.

### Rehabilitation

Patients allocated to either of the two groups will receive the same standardised, written physiotherapy advice detailing the exercises that they need to perform for rehabilitation following their injury. Weight-bearing status will be decided by the treating surgeon. Any other rehabilitation input beyond the written physiotherapy advice will be left to the discretion of the treating clinicians.

### Outcome data

#### Feasibility study

The primary outcome measures for this study are the participant recruitment rate and the completion rate of the EuroQol 5 Dimensions (5 L) score (EQ-5D-5 L) [18] at 4 months post surgery. Measurements that will be made during the trial are summarised in Table 1.

The majority of pre-injury function is recovered in this cohort of patients by 4 months [16, 19]. The outcome measures will, therefore, be collected at this time point. The functional and health-related quality of life outcome data will be collected using the EQ-5D-5 L [18], DEMQoL [20] and DRI [21] at baseline (post injury and, retrospectively, pre injury) and 6 weeks and 4 months post surgery. Where possible patients will complete baseline, 6-week and 4-month questionnaires while in hospital during their initial stay or during routine follow-up appointments, with the help of an appropriate proxy where necessary.

##### Baseline characteristics

Routine baseline characteristics (e.g. age, gender, weight) will be recorded for all participants to describe the nature of the participants.

##### EuroQol 5 Dimensions (5 L) score (EQ-5D-5 L)[18]

The EQ-5D-5 L is a validated, generalised and standardised instrument comprising a Visual Analogue Scale (VAS) measuring self-rated health and a health status instrument, consisting of a five-level response for five domains related to daily activities [18]. Responses to the health status classification system are converted into an overall score using a published utility algorithm for the UK population [22]. A respondent’s EQ-VAS gives self-rated health on a scale where the endpoints are labelled ‘best imaginable health state’ (100) and ‘worst imaginable health state’ (0). EQ-5D-5 L has been validated for use in patients with cognitive impairment where an appropriate proxy may respond to the questions [23] and it can be administered by mail or by telephone. Our recent work has demonstrated it to have excellent measurement properties in comparison with other commonly used disease and region-specific outcome tools in the similar cohort of patients with fragility hip fracture [16, 19]. EQ-5D-5 L scores will be collected at baseline (for pre and post surgery), 6 weeks post surgery and 4-months post surgery.

##### Disability Rating Index (DRI) [21]

The DRI score is a validated self-reported questionnaire. It consists of 12 items specifically related to function of the lower limb. These data will be collected at baseline, 6 weeks and 4 months post surgery in participants who do not have cognitive impairment.

##### Dementia Quality of Life Measure (DEMQoL) [20]

The DEMQoL [20] score is a validated questionnaire specifically designed to assess quality of life in patients with dementia that can be self or proxy-reported. Recently preference based utility scores for a UK population have been published [24].

##### Unit costs for health and social care resources

These will be collected at 6 weeks and 4 months via self-reported patient questionnaires and appropriate proxies. The data collected in the participant questionnaires at each time point will also record indirect costs borne by participants and carers as a result of attending hospital visits; as well as direct non-medical costs (including travel expenses) attributable to their health state.

##### Complications

All complications will be recorded. Complications will be classified as either unrelated to the trial protocol, related systemic complications or related local complications.

##### Radiographic evaluation

Routine anterior-posterior and lateral radiographs of the femur will be assessed for mal-union at 6 weeks post injury. Radiographs will be assessed by an independent researcher at each site.

#### Process evaluation

##### Semi-structured interviews

Patients, carers and staff will be asked to participate in qualitative interviews to discuss their experience of participating in the trial and the intervention. Interviews will be semi-structured, based on the semi-structured interview guide.

##### Grip strength

Grip strength is a measure of muscle strength and gives an indication of sarcopaenia, a predictor of frailty, and will be measured as previously described by Roberts et al. [25].

##### Frailty

The degree of frailty can provide useful predictive information [26], and will be measured using the Rockwood Frailty Scale score [26].

##### Social support

The Medical Outcomes Survey (MOS) social support survey is a brief multidimensional, self-administered social support survey [27].

##### Self-efficacy

Self-efficacy is a measure of an individual’s confidence in their ability to accomplish tasks and overcome problems. Low levels of self-efficacy are associated with less optimal health behaviours [28].

#### Sample size

Data from this feasibility study will be used calculate estimates of the standard deviation of the primary outcome measure (EQ-5D-5 L) to drive a formal power analysis and sample size calculation for the definitive trial. We aim to recruit at seven centres over a total of 52 centre-months. Assuming that the recruitment rate is 1.0 per month per centre and monthly centre counts of patient recruitment numbers are approximately Poisson distributed and independent of one another, then this will allow us to estimate the recruitment rate with a 95% confidence interval of 0.73–1.28 [29].

For the qualitative interviews, convenience sampling will be used to identify potential participants at different time points. The sampling of patients and staff will be reviewed on an ongoing basis throughout the process evaluation, and interviews will continue until data saturation is achieved.

### Analyses

#### Feasibility study

This feasibility study is not powered to formally assess the size of the treatment effect, rather to estimate the recruitment rate. However, the totality of the data collected will be used to assess the feasibility of a definitive, large randomised clinical trial (RCT); recruitment rate being the driver of the feasibility study design on the basis that unless a reasonable recruitment rate can be achieved no formal trial will be possible. The recruitment rate will be estimated based on data collected and a 95% confidence interval determined for this measure. The reasons and patterns of any missing data, loss to follow-up and participant withdrawals will be carefully considered and reported.

If the estimated recruitment rate is such that a definitive trial is feasible then no formal analysis will be undertaken and data from the feasibility study will be locked and carried over into the main (definitive) trial.

If a definitive trial is not feasible, then outcome data will reported in the conventional manner. Baseline demographics (e.g. age, gender, cognitive status) will be compared between groups to ensure approximate balance has been achieved. The main analysis will investigate differences in the primary outcome measure, EQ-5D-5 L [18] score at 4 months, between the two treatment groups (nail and plate) on an intention-to-treat basis. In addition a per-protocol analysis will also be reported and early EQ-5D-5 L status will also be assessed and reported at 6 weeks. Differences between groups will based on a normal approximation for EQ-5D-5 L [16, 19]. Tests will be two-sided and considered to provide evidence for a significant difference if *p* values are less than 0.05 (5% significance level).

The main analyses will be conducted using specialist mixed-effects modelling functions available in the software package R (http://www.r-project.org/) where EQ-5D-5 L [18] data will be assumed to be normally distributed; possibly after appropriate variance-stabilising transformation. The primary focus will be the comparison of the two treatment groups of patients, and this will be reflected in the analysis which will be reported together with appropriate diagnostic plots that check the underlying model assumptions. Results will be presented as mean differences between the trial groups, with 95% confidence intervals.

Secondary analyses will be undertaken using the above strategy for approximately normally distributed outcome measures such as DRI [21]. For dichotomous outcome variables, such as complications related to the trial interventions, mixed-effects logistic regression analysis will be undertaken with results presented as odds ratios (and 95% confidence intervals) between the trial groups.

### Process evaluation

The evaluation will focus on investigating the fidelity and quality of implementation, clarifying the causal mechanisms and identifying the contextual factors which might be important in variation of outcome. We will work with patients and with stakeholders to define a logic model for the complex intervention. From this model we will develop the key assumptions which underpin the intervention within the settings of the feasibility study. We will adopt a mixed-methodology approach in order to address the key uncertainties around the complex perioperative and rehabilitation intervention.

Interviews with patients, surgeons and other staff will be transcribed verbatim. The material will be organised into themes, using inductive coding. Using a constant comparative approach, these themes and their sub-themes will be used to produce a coding framework. The relationship between themes and sub-themes will be illustrated in a thematic map.

### Health economic evaluation

The feasibility of a future, definitive economic evaluation of treatment with modern intramedullary nails or anatomical locking plates for fragility fractures of the distal femur will be investigated. Unit cost data will be obtained from national databases such as the *British National Formulary* (BNF) and PSSRU Costs of Health and Social Care [30]. EQ-5D-5 L evaluations be collected at baseline (pre-injury status and current injury status), and follow-up will be used to estimate quality-adjusted life years (QALYs) in any future trial-based economic evaluation.

### End of the study

All patients will be followed up to collect data on their status at 4 months post operation. If data cannot be collected within 3 months of this point, follow-up data will not be collected. After the 4-month time point participants will have no further study-specific follow-up.

### Study oversight

The conduct of the study is overseen by a Data Safety Monitoring Committee comprised of independent experts and a Trial Steering Committee with a minimum of 75% independent members, including experts and a patient representative. The trial was sponsored by the University of Oxford (contact: Heather.House@admin.ox.ac.uk).

## Discussion

This is the protocol for a feasibility study conducted prior to any future definitive trial. The objectives of any planned future trial are to:

1. Quantify and draw inferences on observed differences in health-related quality of life at 4 months between the study intervention groups (nail versus plate)
2. Quantify and draw inferences on observed differences in functional status at 4 weeks, 4 months and at 12 months
3. Quantify and draw inferences on observed differences in the radiological outcomes of non-union, mal-alignment and shortening
4. Determine the complication profile associated with nail fixation versus locking-plate fixation in the first year after the injury
5. Investigate, using appropriate statistical and economic analytical methods, the resource use, costs and comparative cost-effectiveness of nail fixation versus locking-plate fixation

The estimates of participant recruitment rate and proportion of data completion will be coupled with outputs from the process evaluation to make a final decision regarding feasibility. The recruitment criteria, against which a decision to determine the feasibility of a definitive trial can be judged, are given below. If these criteria are not met a full trial based upon the design of this feasibility study is unlikely to be pursued. These criteria are:

1. An average recruitment rate of 1.0 participants per month per centre
2. Appropriate recruitment performance across all feasibility sites

### Trial status

At the time of manuscript submission, trail recruitment was ongoing.

## Abbreviations

DEMQoL: Dementia Quality of Life Measure
DRI: Disability Rating Index
EQ-5D-5 L: EuroQol 5 Dimensions Score
RCT: Randomised clinical trial
TrAFFix: Trial of Acute Femoral Fracture Fixation
